# Sternal osteomyelitis caused by *Aspergillus fumigatus* years after coronary artery bypass grafting: A case report

**DOI:** 10.1016/j.idcr.2022.e01638

**Published:** 2022-11-01

**Authors:** Austin Hingtgen, Rishav Aggarwal, Shreya Avilala, Azmath Mohammed, Rosemary Kelly

**Affiliations:** a University of Minnesota Medical School, 420 Delaware St. SE, Minneapolis, MN 55455, USA; b Division of Cardiothoracic Surgery, Department of Surgery, University of Minnesota Medical School, 420 Delaware St. SE, MMC 207, Minneapolis, MN 55455, USA

**Keywords:** Aspergillus, Osteomyelitis, Sternal, CABG, Cardiac

## Abstract

Sternal infection after cardiac surgery is an infrequent post-operative complication. *Aspergillus* sternal osteomyelitis is a rarity. We review the case of a 77-year-old man with invasive aspergillosis of the sternum and left costal cartilage 23 years after undergoing cardiac surgery. The patient promptly underwent surgical irrigation and debridement, followed by antifungal therapy. Clinical suspicion of sternal fungal infection should be high in patients with mediastinitis with a history of cardiac surgery. Treatment should be prompt.

## Highlights

• *Aspergillus* sternal osteomyelitis is a rare complication after median sternotomy.
• Invasive aspergillosis rarely causes osteomyelitis several years after sternotomy.
• Treatment for sternal osteomyelitis should begin upon suspicion of infection.
• Irrigation and debridement are necessary for sternal osteomyelitis treatment.
• Voriconazole therapy is effective for non-resistant *Aspergillus* osteomyelitis.

## Introduction

*Aspergillus* species are fungi found in organic matter that form acute-angle branching septate hyphae and cause life-threatening infection in immunocompromised individuals. Often contracted through direct inoculation or inhalation of *Aspergillus* mold spores, a wound or respiratory infection may become invasive in immunocompromised individuals, leading to systemic infection [1]. Rarely, *Aspergillus* infection presents as sternal osteomyelitis [2]. We present a case of *Aspergillus fumigatus* sternal osteomyelitis years after sternotomy for coronary artery bypass (CABG) surgery.

## Case

This is a case of a 77-year-old man with history of Type 1 Diabetes Mellitus (DM), Rheumatoid Arthritis (RA), Heart Failure, Chronic Obstructive Pulmonary Disease (COPD), and Coronary Artery Disease who underwent 2-vessel CABG in 1997. He was on rituximab therapy for RA and budesonide/formoterol for COPD. His rituximab regimen began with two initial doses (1000 mg each) two weeks apart in October 2019. Previously, his RA was controlled with etanercept, hydroxychloroquine, and leflunomide, but this regimen was discontinued due to the development of vasculitis lesions. He was subsequently transitioned to rituximab therapy for persistent arthritis and pulmonary symptoms related to his RA. His last dose of rituximab was in May 2020.

In February 2020, the patient had a myocardial infarction with left ventricular thrombus requiring an 11-day hospitalization for warfarin anticoagulation. Subsequently, 4 months later, the patient fell and broke left-sided ribs and sustained a T12 vertebral fracture. He had a chest CT that incidentally showed evidence of erosive and destructive changes to the sternum consistent with osteomyelitis, but no therapy was started as he was asymptomatic. This CT also demonstrated new areas of clustered nodularity in the right lung, and to a lesser extent in the left lower lobe. The left lower lobe consolidation appeared to be a cavitary lesion, but it was unclear if this represented a true cavity nodule or an area of bronchiectasis. There were no ground-glass opacities noted. The scan also showed a chronic left pneumothorax from trauma in 2014. These imaging finding were complicated by the patient’s rheumatoid arthritis, as the condition causes diffuse lung nodules.

**Fig. 1:**
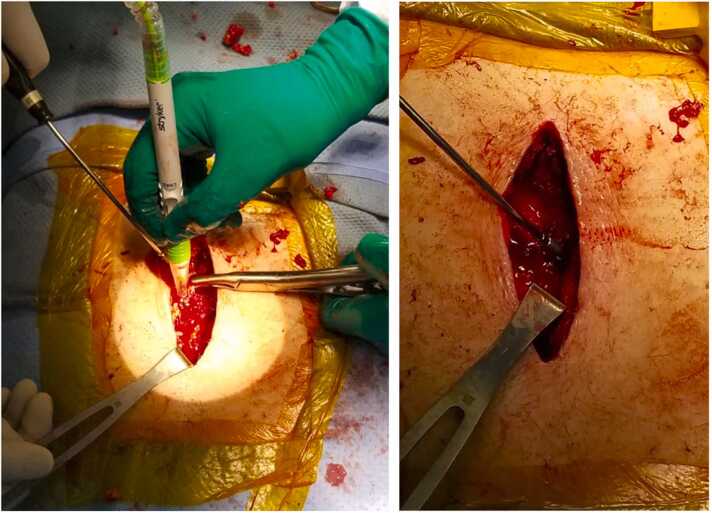
Intraoperative photographs showing irrigation and debridement (ID) of sternal wound (left) and the open sinus following ID and removal of infected cartilage and sternum (right).

**Fig. 2:**
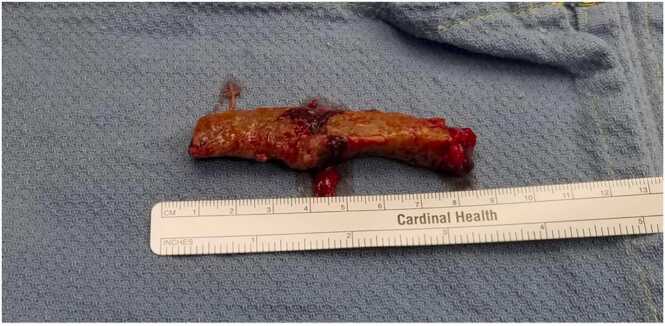
Complete left costal cartilage “cast” displaying chronic destruction.

**Fig. 3:**
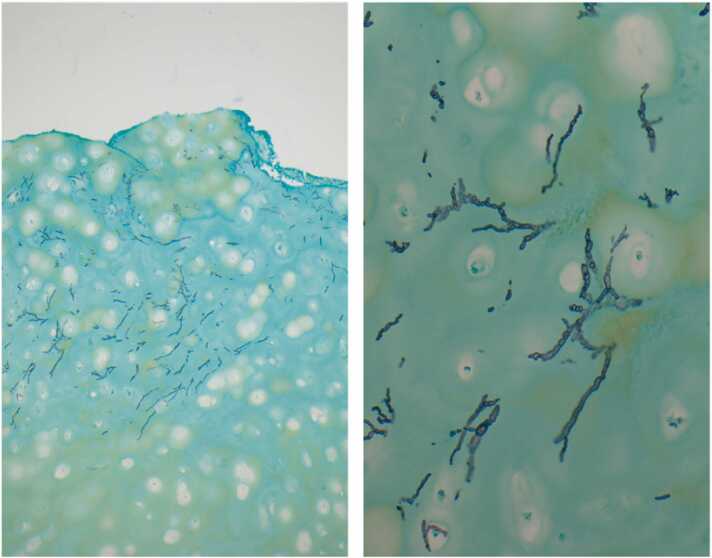
Histopathology of removed costal cartilage displaying acute angle branching, narrow, septate hyphae of *Aspergillus fumigatus* (10x left and 50x right; Grocott-Gomori's methenamine silver stain).

Two months later he presented with chest tenderness and pruritic rash at his old sternotomy scar. On exam, there was a 2 cm area of erythema with yellow-brown drainage at the inferior portion of his otherwise well healed sternal scar. Both C-Reactive Protein (CRP) and Erythrocyte Sedimentation Rate (ESR) were elevated (98.14 mg/L and >130 mm/hr respectively). Contrast chest CT showed increased thickening of the soft tissues anterior to the sternum and in the left anterior chest wall with a new 4 cm (transverse) by 9 cm (craniocaudal) parasternal phlegmon that extended to the skin surface. All findings were alarming for osteomyelitis of the sternum and ribs. While waiting for culture and histopathology results, a 1,3-ß-D Glucan test returned as elevated (>500 pg/ml) and the *Aspergillus* (Galactomannan) antigen index result was 0.19 (Ref: <0.50). KOH fungal prep of the drainage showed acute angle branching septate hyphae and the fungal prep displayed 2 + *Aspergillus fumigatus* sp. complex based on micro and macro pathology, including (sub)globose vesicles and uniseriate aspergilla with chains of conidia. This indicated deep seated invasive *Aspergillus fumigatus* conidia infection. He underwent irrigation and debridement, inferior sternectomy, and removal of wires (Fig. 1). The left cartilage segments for ribs four through seven were removed in complete “casts” with evidence of cobblestoning and chronic destruction (Fig. 2). Intraoperative wound KOH and cultures were consistent with previous cultures. Histology stained with Grocott-Gomori's Methenamine Silver displayed acute-angle branching septate hyphae (Fig. 3). Susceptibility testing for *Aspergillus* was not available at the institution that this case took place, so voriconazole was chosen as empiric therapy.

The patient was given an initial loading dose (400 mg Q12Hr) of IV voriconazole for the first day, then was transitioned to oral voriconazole (300 mg BID). His serum voriconazole level 4 days later was 8.6 mcg/ml. Our target range for serum voriconazole level was a trough level of 2.0 – 5.5 mcg/ml as this is reported as therapeutic range for treatment. On day 12 post-loading dose, the patient’s alkaline phosphatase was elevated at 3358 U/L. It was decided to decrease his dose of voriconazole therapy to 200 mg PO BID. Six days later his Voriconazole level was measured at 1.0 mcg/ml, so his treatment was increased to 250 mg PO BID. One month after initiation of treatment, the patients voriconazole level was 1.4 mcg/ml and his alkaline phosphatase was 879 U/L, so his treatment dose was titrated for the last time to 300 mg PO in the morning and 250 mg PO in the evening. The patient was discharged to a community living center (CLC) where he received wound vac care three days a week and his labs were continually monitored. His serum voriconazole levels were measured twice more over the next month and a half at 3.1 mcg/ml and 5.1 mcg/ml.

Two and a half months after the initiation of therapy, the patient was re-admitted to the hospital for several days of band-like chest pain and fever. He was found to have new nodular right upper lobe opacities; his c-reactive protein was 231 and his pro-calcitonin was 1.61, but there was no leukocytosis. A respiratory panel was positive for pseudomonas, and he was treated with Zosyn for 1 day while admitted. On discharge, the patient was prescribed ciprofloxacin 750 mg Q12 to complete a 10-day course of antibiotic treatment.

At the 3-month postoperative visit he had been discharged from the CLC with his sternal wound clean, dry, and intact without any signs of infection. The plan was to continue Voriconazole therapy (300 mg PO QAM and 250 mg PO QPM) for 3 more months, then discontinue and monitor. CT scan was scheduled for 6- and 9-month post-operative follow-up. The patient’s last follow up appointment was in December 2020, 4 months post-op, and there were no signs of continued infection. He subsequently died that same day at an outside facility due to suspected unrelated causes.

## Discussion

This 77-year-old man with COPD, RA, and DM, on rituximab and insulin, presented with sternal osteomyelitis caused by *Aspergillus fumigatus* beneath the previous incision site from CABG 23 years earlier. Fungal infections causing sternal osteomyelitis are rare with *Aspergillus* species being the most identified fungi 1, 2. These are mostly seen in immunocompromised patients and may likely increase as the number of cases in immunocompetent patients is slowly on the rise [3]. This patient was undergoing immunosuppressive therapy and had a history of COPD and DM, making him more vulnerable to developing an *Aspergillus* infection. Although, at the time of diagnosis the patient’s Hgb A1c was 7.5 and his diabetes was controlled with an insulin aspart pump, making DM less likely contributory to his infection.

This case is unusual as most sternal infections happen within a few months of initial sternotomy [2]. There are three mechanisms by which osteomyelitis caused by *Aspergillus* generally occurs. These include hematogenous spread from needle injection or pulmonary infection, spread from pleuropulmonary disease, or infection of a site of trauma or surgery directly. The progression of this infection was likely triggered by the fall while on anticoagulation causing an internal hematoma that became seeded with *Aspergillus*. Although initially only the sternum was involved, the infection progressed within 2 months to involve several cartilage and soft tissue. Prompt treatment included surgical debridement and voriconazole therapy. In this case, the infection was initially dismissed by infectious disease due to the individuals lack of symptoms. Given the subclinical nature of *Aspergillus* osteomyelitis presentation, one should have a high clinical suspicion, and antifungal treatment and surgery should be completed as soon as possible [4].

Treatment of Aspergillus osteomyelitis commonly consists of surgical debridement plus antifungal therapy [1]. Asare et al. reviewed nine cases of sternal or costal *Aspergillus* osteomyelitis. Five patients were treated with amphotericin B initially, then switched to itraconazole for a total of 10–15 weeks of therapy. All of them were cured of infection at the end of treatment. Three patients were treated with voriconazole for a total treatment time of 8 weeks to 6 months. All three patients were cured of infection after treatment. One patient died of an unrelated cause after 30 days of itraconazole treatment. Seven of the nine patients in Asare et al.’s review underwent surgical debridement, and no patients died due to their *Aspergillus* osteomyelitis [3].

Azoles are the preferred agents for invasive aspergillosis in general, particularly voriconazole. Posaconazole and Isavuconazole are alternatives to voriconazole with proven efficacy in randomized trials 5, 6. Amphotericin B is also indicated as primary and salvage therapy but is preferred second to azoles in clinical practice due to more frequent severe adverse events [1]. Furthermore, voriconazole is proven superior to Amphotericin B in the treatment of invasive aspergillosis with improved survival 1, 3, 4. It is suggested that treatment with voriconazole should last a minimum of 8 weeks and in some cases greater than 6 months [1]. During voriconazole treatment, 11 weeks post-op, this patient's sternal wound was clear of any sign of infection, which is supportive of the ability to manage sternal osteomyelitis caused by invasive aspergillosis with a combination of surgical debridement and voriconazole therapy.

## Ethical approval

Obtained

## Consent

Written informed consent was obtained from the patient for publication of this case report and accompanying images. A copy of the written consent is available for review by the Editor-in-Chief of this journal on request.

## Funding

None.

## CRediT authorship contribution statement

**Austin Hingtgen**: Conceptualization, methodology, formal analysis, investigation, resources, data curation, writing – original draft, writing – review & editing, visualization, **Rishav Aggarwal**: Validation, formal analysis, writing – review & editing, supervision, **Shreya Avilala**: Investigation, writing – original draft **Azmath Mohammed**: Conceptualization, validation, writing – review & editing, supervision, **Rosemary Kelly**: Conceptualization, methodology, validation, writing – review & editing, supervision, project administration.

## Conflict of interest

There are no conflicts of interest.
